# Hyperoxia-induced lens damage in rabbit: protective effects of N-acetylcysteine

**Published:** 2009-12-31

**Authors:** Ping Wang, Xiao-Cui Liu, Hong Yan, Ming-Yong Li

**Affiliations:** 1 Department of Ophthalmology, Tangdu Hospital, Fourth Military Medical University, Xi’an, China; 2 Department of Stomatology, No. 451 Hospital of PLA, Xi’an, China

## Abstract

**Purpose:**

To investigate the efficacies of different concentrations of N-acetylcysteine (NAC) in preventing hyperoxia-induced lens opacification and changes to biochemical parameters in organ-cultured rabbit lenses.

**Methods:**

Thirty-six lenses from adult rabbits were divided into the control group (group A), the hyperoxia-exposed group (group B), and the hyperoxia-exposed, NAC-treated groups: 5 mM NAC (group C), 10 mM NAC (group D), 20 mM NAC (group E), and 40 mM NAC (group F). Groups B–F were incubated with hyperoxia (pO_2_>80%) for 4 h per day for 7 d. Lens transparency, histology, and enzymatic activities were measured after incubation.

**Results:**

Gross examination of these lenses revealed some severe cortical opacification in group B, and moderate cortical opacification in the lenses of groups C and D. There was minimal cortical opacification in groups A, E, and F. The activities of Na, K-ATPase, and catalase were significantly (p<0.05) lower in group B (38.2%) than in group A (39.9%). It was also lower in group E and F lenses (p<0.05), which had higher levels of NAC-protected enzymes. The glutathione and water-soluble protein content were significantly lower in group B lenses than in group A, E, or F lenses (p<0.05). However, there was no difference between group E and F lenses (p>0.05).

**Conclusions:**

The present data suggests that NAC (20 mM–40 mM) significantly prevented experimental lenses’ hyperoxia-induced cortical opacification, indicating NAC’s potential role in protecting lenses against cataracts induced by high oxygen levels.

## Introduction

Cataract is a progressive opacity of the lens of the eye that impairs vision. It is the leading cause of human blindness worldwide, accounting for more than half of all blindness. The prevalence of cataract in developing countries is much greater than in developed ones [1]. Although the causative factors of cataract are many, and its etiology is still unclear, it has been linked to oxidation [2]. It plays a major role, not only in age-related cataract, but also in cataract after vitrectomy and hyperbaric oxygen (HBO) therapy [3-5]. Changes in the lens under hyperbaric oxygen have been used as a model for cataract research [6]. We investigated the damaging effects of hyperoxia on lens transparency and biochemistry, and the possible protective effect of N-acetylcysteine (NAC) against this damage.

The cornea and retina are highly oxygenated. However, the partial pressure of O_2_ in the center of the lens is normally low, approximately 10 mm Hg, or 1% [7], due in part to metal-catalyzed reactions of the molecule with glutathione (GSH) [8]. Sakaue et al. [9] measured vitreous oxygen tension in the human vitreous body using a paleographic oxygen electrode. The mean oxygen tensions of the anterior peripheral vitreous body, central vitreous body, and posterior vitreous body were 16.7±3.7 mm Hg, 15.9±2.8 mm Hg, and 19.9±4.8 mm Hg, respectively, while the preretinal oxygen tension of the detached retina was 30.0±4.8 mm Hg. The mean oxygen tension around the lens was almost equal to that of the posterior vitreous body. Much of the O_2_ that enters the anterior side of the lens from the aqueous humor is consumed by mitochondria located in the lens’s anterior epithelium [10,11]. During oxidative phosphorylation, some electron leakage to oxygen occurs, forming superoxide. Mitochondrial production of superoxide increases with age. Superoxide may diffuse throughout the lens, that is, via the lens membranes. This process could contribute to elevated hydrogen peroxide (H_2_O_2_) in the nuclei of old lenses. Hydrogen peroxide appears to contribute to lens damage, and H_2_O_2 _alone is not sufficient. H_2_O_2 _itself is not a strong oxidant; however, the hydroxyl radicals it produces in the presence of ferrous ions, through the Fenton reaction, are highly reactive, and can damage proteins, lipids, and nucleic acids, as well as small molecules [12-15]. It also induces expression of the proto-oncogenes (c-jun, c-fos, and c-myc in rabbit-lens epithelial cells), which may regulate lens crystallin genes and other genes containing activating protein-1 (AP-1) binding sites [16,17]. Elimination of hydrogen peroxide is critical in reducing oxidative stress. Glutathione peroxidase and catalase serve this purpose.

GSH plays a key role in the protection against oxidative stress, which is quantitatively the most important endogenous rechargeable antioxidant, and which functions as an essential antioxidant for maintaining the tissue’s transparency [18]. GSH is synthesized in the cortex, and intercepts those reactive intermediates. If H_2_O_2_ is formed, GSH will be oxidized to GSSG. In the presence of protein sulfhydryl (-SH) groups, GSSG can cause protein thiolation [14]. However, glutathione reductase (GR) reduces GSSG to GSH with electrons donated from the reduced form of nicotinamide-adenine dinucleotide phosphate (NADPH), allowing GSH to protect protein thiols from oxidative damage, and preventing protein cross-link formation [19].

Catalase is a key enzyme that plays a role in intracellular lens defense mechanisms. It converts H_2_O_2_ to H_2_O to reduce oxidative damage [14], and the catalase (CAT) of eye tissues regulates the endogenous H_2_O_2_ in eye humors at the physiologic level, which also protects the cation pump [15,20]. Na, K-ATPase is responsible for maintaining normal physiologic concentrations of sodium and potassium in lens cells, and for maintaining the ionic balance of the cells [21,22]. Decreasing both the GSH content and the catalase and Na, K-ATPase activities may increase oxygen-associated cellular damage, as well as the rate of cataract formation. In the present study, we examined GSH content and the activities of Na, K-ATPase and catalase in lenses exposed to hyperoxia, both with and without various concentrations of NAC in the culture medium.

Since GSH is not effectively transported into cells, NAC, a precursor of cysteine, and thus of GSH, has been used effectively to replenish intracellular GSH stores directly and conveniently [23]. It is an excellent source of sulfhydryl (-SH) groups, and is converted in the body into metabolites capable of stimulating GSH synthesis. Jain’s study showed that NAC supplementation might be helpful in slowing the oxidation of rabbit-lens proteins in vitro [24]. Shattuck et al. [25] also concluded that NAC prevents oxidant stress during hyperoxic exposure, most likely by supplying cysteine as a precursor for GSH synthesis. Our previous study found that NAC eyedrops could prevent streptozotocin-induced diabetic cataract in rats [26]. Injection of NAC into the vitreous cavity protected the lens against some biochemical changes induced by the increased oxygen after vitrectomy [27]. Rathbun et al. [28] showed that L-cysteine prodrugs were effective in preventing naphthalene-induced cataract, and in maintaining near-normal hepatic GSH levels. In addition, Li et al. [16,17] have reported that NAC could upregulate the mRNAs of both c-jun and c-fos, and enhance the transactivity of activating protein-1 (AP-1). Therefore, it is possible that NAC may have protective effects against lens damage by hyperoxia. The purpose of the present study was to investigate the efficacies of different concentrations of NAC in preventing the changes caused by hyperoxia to rabbit lenses under organ-culture conditions.

## Methods

### Materials

N-acetylcysteine (NAC) was purchased from Sigma Chemical Company (Beijing, China). Rabbits were provided by the Animal Laboratory of the Fourth Military Medical University (Xi’an, China). Protein and enzyme quantification kits were obtained from Jiancheng Biology Company (Nanjing, China). All other chemicals and solvents were analytic grade and obtained commercially from local companies (Xi'an, China).

### Experimental groups

New Zealand rabbits (1.5–2.0 kg, 4 months old) were killed by an overdose of sodium pentobarbital. The lenses (whose weights were approximately 300 mg each) were removed from eyes that had been excised by a posterior approach. The lenses were at once immersed in Dulbecco’s Modified Eagle Medium (Sigma, St. Louis, MO), which contained calf serum (10%), streptomycin (5×10^4^ U/l), and penicillin (5×10^4^ U/l), to prevent microbial contamination.

All the lenses were examined after 8 h pre-experimental incubation. Only the intact clear lenses were chosen for experimentation, and those with any lens defects were rejected. In the experiments, each lens was cultured in 5 ml of medium having a liquid-gas interphase surface area of 3.6 cm^2^. Prior to culture, the medium was sterilized by passage through a 0.2 μm filter [6]. The selected clear lenses were divided into six groups:

A: Control group: six lenses were incubated in the medium with an atmosphere of 95% room air and 5% CO_2_.
B: Hyperoxia exposed group: six lenses were incubated in the medium and treated daily with oxygen (>80%) at 1.0 atm (atmospheric pressure) for 4 h.
C: 5 mM NAC-treated group: six lenses were incubated in the medium containing 5 mM NAC and treated daily with oxygen (>80%) at 1 atm for 4 h.
D: 10 mM NAC-treated group: six lenses were incubated in the medium containing 10 mM NAC and treated daily with oxygen (>80%) at 1 atm for 4 h.
E: 20 mM NAC-treated group: six lenses were incubated in the medium containing 20 mM NAC and treated daily with oxygen (>80%) at 1 atm for 4 h.
F: 40 mM NAC-treated group: six lenses were incubated in the medium containing 40 mM NAC and treated daily with oxygen (>80%) at 1 atm for 4 h.

All lenses were subjected to gross morphological examination daily. In addition, after the 7 d incubation period, quantitative analyses of enzyme activities were performed for the lenses of all groups. In a preliminary experiment, five different concentrations of NAC (5, 10, 20, 40, and 100 mM) were chosen to examine the side effects of NAC, by itself, on lenses.

### Hyperoxia treatment

**Figure 1:**
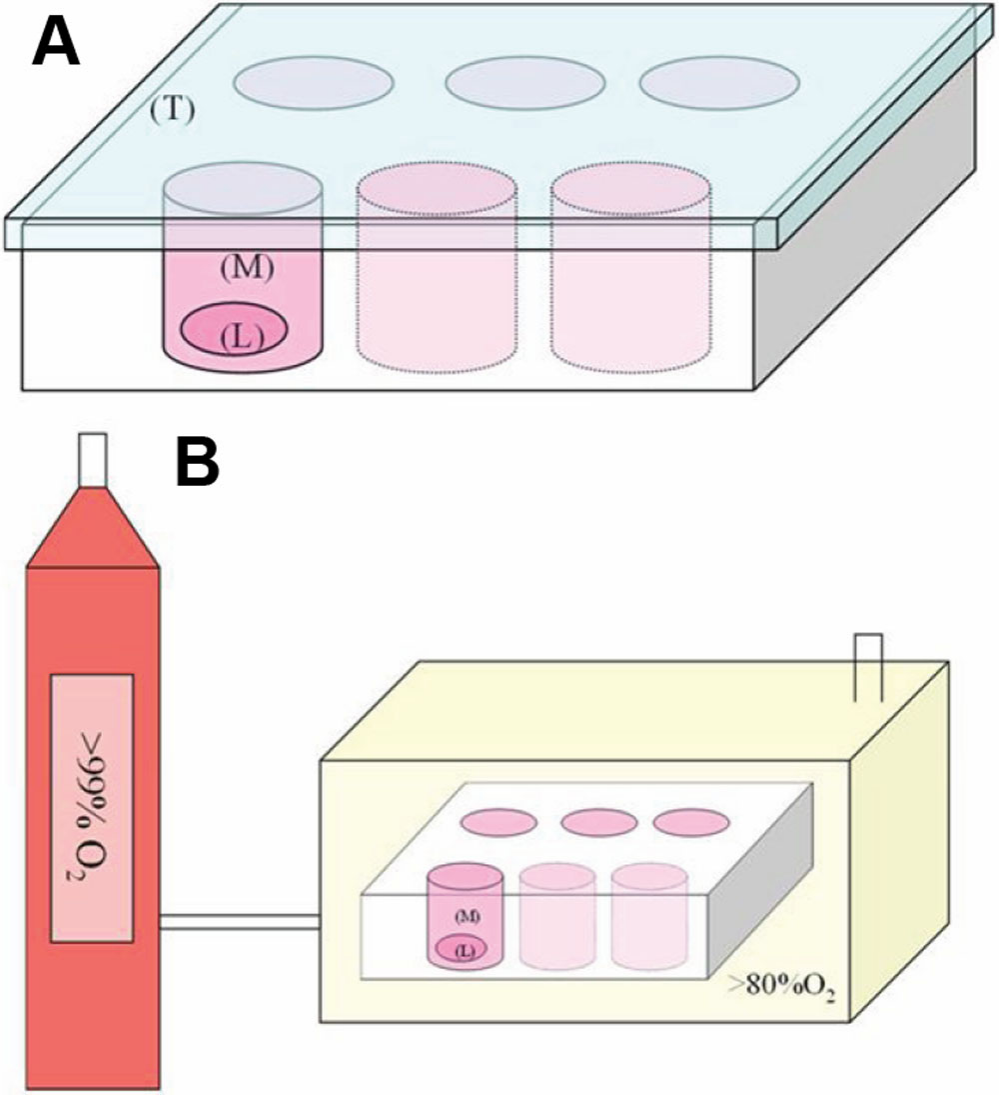
Cultured lenses exposed to hyperoxia in a sealed chamber. **A**: Lens culture chamber scheme: Lens (L) is totally immersed in culture medium (M). The chamber is placed vertically, with a covered top (T). **B**: Sealed chamber scheme: The cylinder supplied oxygen (99%) into the sealed chamber containing the culture chambers without a covered top, and the oxygen saturation inside the sealed chamber was kept above 80%.

The cultured lenses were exposed to hyperoxia in a sealed chamber (Figure 1). The temperature inside the chamber was maintained at 37 °C by keeping the vessel in a temperature-controlled water bath. The concentration of oxygen was raised to 80% over a 5 min period and then maintained for 4 h daily. The pressure in the chamber was kept at 1.0 atm. The culture medium was changed daily before the hyperoxia test, and all the work was completed under a superclean bench to prevent microbial contamination. During the test, oxygen saturation inside the sealed chamber was monitored by an oxygen meter (CY-12C; Hangzhou, China) and kept constant.

### Examination of the lenses

**Figure 2:**
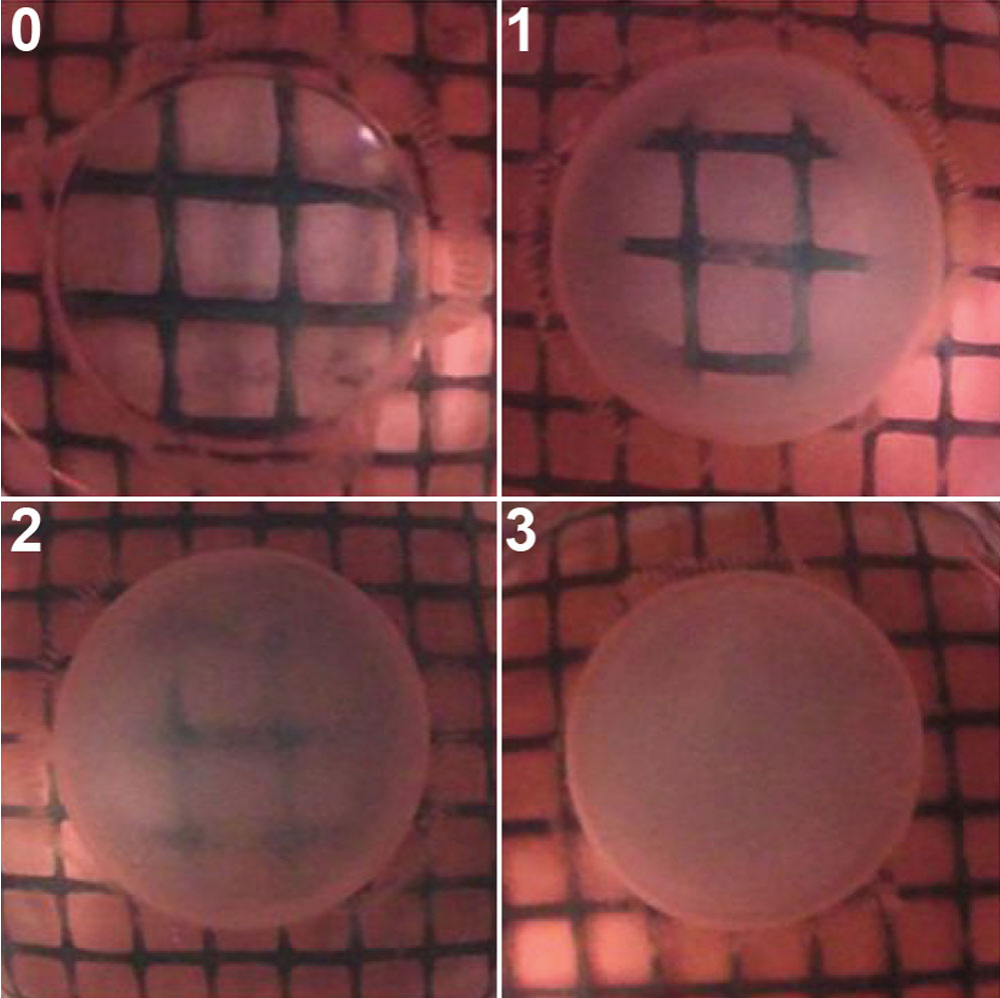
Standard photographs to illustrate the system for grading lenses. Grade 0 means the absence of opacification and the gridlines clearly visible; grade 1 means slight degree of opacification, the gridlines under the lens are mininal clouding and still visible; grade 3 means extensive thick opacification involving in the entire lens, the gridlines are total clouding and not seen at all.

Lenses were examined by eye, as well as under the magniﬁcation of a dissecting microscope against a background of black gridlines. The degree of opaciﬁcation was assessed according to Geraldine et al. [29], described as follows: grade 0, absence of opacification (gridlines clearly visible); grade 1, slight degree of opacification (minimal clouding of gridlines and gridlines still visible); grade 2, diffuse opacification involving almost the entire lens (moderate clouding of gridlines and gridlines faintly visible); grade 3, extensive thick opacification involving the entire lens (total clouding of gridlines and gridlines not seen at all; Figure 2). A double-blind method was used for classifying the degree of lens opaciﬁcation.

### Protein determination

Protein concentration was determined by the Coomassie brilliant blue method by using a protein assay kit. Each lens was ground in 0.9% physiologic saline (1 g:9 ml), homogenized using a handheld homogenizer for 15 min over ice, and then centrifuged (3,000 rpm, 10 min) in Eppendorf tubes. The clear supernatant was used for water-soluble protein determination, according to kit instructions (Jiancheng, China).

### Glutathione determination

The content of GSH in each lens was determined with 5–5′ dithio-bis-(2-nitrobenzoic acid), following centrifugation (3,500 r/min) in 2 ml of 10% trichloroacetic acid for 10 min, and measured using the colorimetric method at 25 °C and at 412 nm [30].

### Enzyme activities

#### Assay of Na, K-ATPase activity

Na, K-ATPase activity was measured by adding 0.1 ml of the clear supernatant of homogenate to 0.6 ml buﬀer containing 100 mM NaCl, 20 mM KCl, 5 mM MgCl_2_, 3 mM ATP, and 50 mM Tris (pH 7.4). After 15 min preincubation at 37 °C, ATP was used as the substrate. The liberated inorganic phosphate was estimated by spectrophotometric estimation [31]. Ouabain was used as a specific blocker of Na, K-ATPase activity. The ouabain-sensitive ATPase activity was estimated and expressed as micromoles of inorganic phosphate released per mg protein per h.

#### Assay of catalase activity

CAT activity was determined by the method of Beers and Sizer [32] with modification in 1952, by spectrophotometric recording of the cleavage of H_2_O_2_ at 240 nm. The reaction mixture contained 0.5 ml of 23 mM H_2_O_2_ in 1.0 ml of 0.05 M phosphate buffer (pH 7.0). The amount of H_2_O_2_ was far in excess of that of GSH. For this reason, GSH peroxide activity (for which GSH is an obligatory substrate) was assumed to be negligible, compared to catalase (whose only substrate is H_2_O_2_). The activity of catalase was expressed as units/mg protein (one unit was the amount of enzyme that used 1 mM of H_2_O_2_/min).

#### Assay of glutathione reductase activity

Glutathione reductase (GR) activity was measured according to the procedure of Bergmeyer in his book in 1963 [33]. The reaction was initiated by the addition of 20 μl lens homogenate. Oxidized GSH (glutathione disulfide [GSSG]), was reduced to GSH catalyzed by GR, with NADPH as the cofactor. One unit was equivalent to the oxidation of 1 mM of NADPH per min.

### Statistical analysis

One-way analysis of variance (ANOVA) was used for testing statistical significances among groups. The median calculation of the lens opacity for each group was analyzed by using the Wilcoxon rank sum test. A p value of <0.05 was considered significant. All the data were analyzed using the SPSS 13.0 statistical package.

## Results

### Grading of the lenses

In a preliminary experiment, the pH value in the 100 mM NAC medium was decreased to approximately 5 after 24 h incubation under normal oxygen conditions, and the lenses became opaque. However, lenses in the other four groups with NAC (5, 10, 20, and 40 mM) remained clear, and the pH value did not change significantly (data not shown). Therefore, we chose these four concentrations of NAC for the current study and the medium was changed daily before the hyperoxia insult.

**Figure 3:**
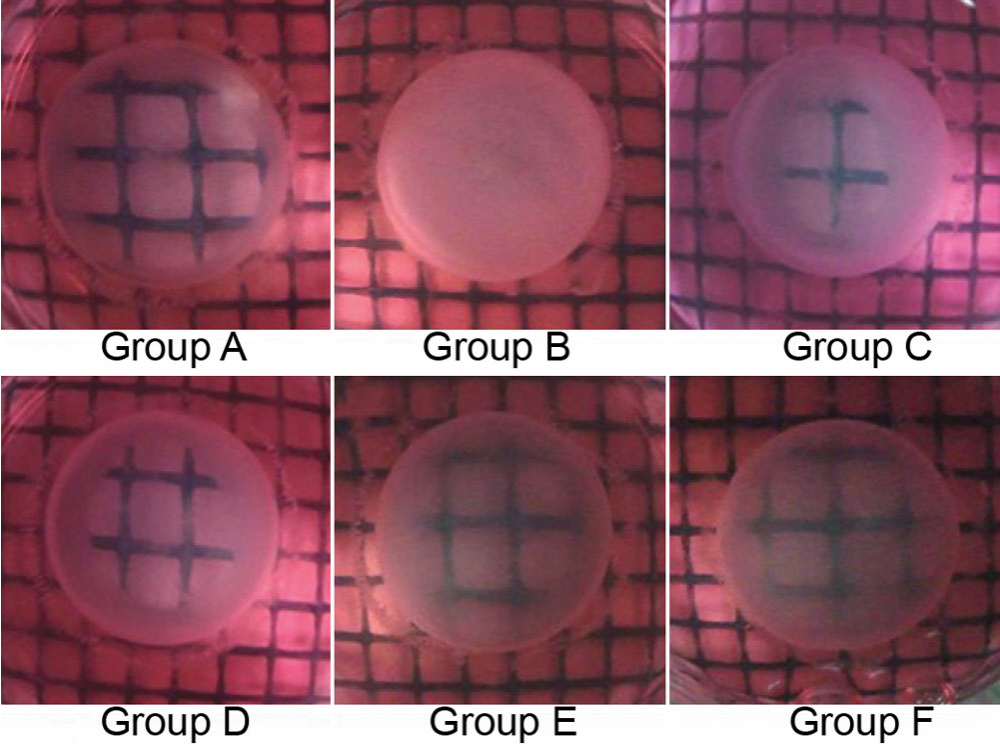
Lenses’ optical clarity after 7 d incubation. Lenses in the control group (A), and those in the 5 mM and 10 mM NAC-treated groups (C and D), were in grade 1 with a slight degree of opacification (minimal clouding of gridlines and gridlines still visible). Lenses in group B were totally opaque (grade 3, gridlines not seen at all). Most lenses in 20 mM and 40 mM NAC-treated groups were kept transparent (grade 0). Group A was the control group, group B was the hyperoxia-exposed rabbit lenses group, groups C-F were the hyperoxia-exposed, NAC-treated groups: 5 mM NAC (group C), 10 mM NAC (group D), 20 mM NAC (group E), and 40 mM NAC (group F)

In the present study, except for two lenses in group B, no lenses had detectable opacification under examination by dissecting microscope after 3 d. The median lens opacity is presented in Table 1. However, on the fourth day, cortical opacification of lenses was observed in group B. More than 50% of these lenses showed slight cortical opacification, whereas lenses in other groups remained clear (p<0.05; Table 1). After 7 d, all six lenses in group B exhibited total cortical opacification (Grade 3). In contrast, only a few lenses revealed lens opacification in groups C and D, with minimal opacification in groups A, E, and F (p<0.01; Figure 3). These observations indicate that NAC delayed the progression of hyperoxia-induced cortical opacification of lenses.

**Table 1:** Intensity of opaciﬁcation in the isolated rabbit lenses (n=36, M).

**Group**	**Degree of opaciﬁcation (0–3)**						
	**1 day**	**2 d**	**3 d**	**4 d**	**5 d**	**6 d**	**7 d**
A	0	0	0	0	1	1	1
B	0	0	0	1*	2*	3**	3**
C	0	0	0	0	1	1	1
D	0	0	0	0	1	1	1
E	0	0	0	0^#^	1^#^	1^##^	1^##^
F	0	0	0	0^#^	1^#^	1^##^	1^##^

Values are expressed as medians, Median representation: 0, absence of opacification (gridlines clearly visible); 1, slight degree of opacification (minimal clouding of gridlines and gridlines still visible); 2, diffuse opacification involving almost the entire lens (moderate clouding of gridlines and gridlines faintly visible); 3, extensive thick opacification involving the entire lens (total clouding of gridlines and gridlines not seen at all). Statistical analyses were performed by using Wilcoxon rank sum test. The asterisk indicates a p<0.05 B versus A, the double asterisk indicates a p<0.01 B versus A, the sharp (hash mark) indicates a p<0.05 B versus E and F, and the double sharp indicates a p<0.01 B versus E and F. Group A was the control group, group B was the hyperoxia-exposed rabbit lenses group, group C-F were the hyperoxia-exposed, NAC-treated groups: 5 mM NAC (group C), 10 mM NAC (group D), 20 mM NAC (group E), and 40 mM NAC (group F).

### Protein determination

**Figure 4:**
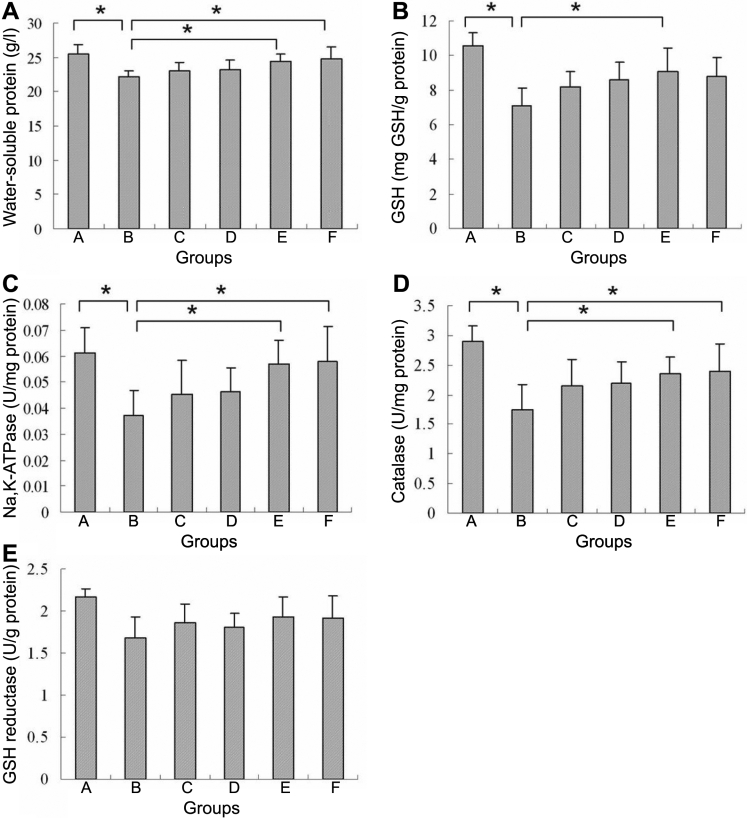
Effects of hyperoxia in rabbit lenses after 7 d incubation compared with controls and the NAC treatment. **A**: The water-soluble protein in rabbit lenses (mean±SD). Lenses exposed to hyperoxia (group B) had a significant reduction in the water-soluble protein (*p<0.05). Compared with the hyperoxia-exposed group (group B), the level of water-soluble protein was increased dramatically in the 20 mM and 40 mM NAC-treated groups (*p<0.05). No significant differences were observed between any other two groups. **B**: The effect of NAC on GSH content of rabbit lenses (mean±SD). Lenses exposed to hyperoxia (group B) had a significant reduction of GSH (*p<0.05). A statistically significant difference was observed between the hyperoxia group (group B) and the 20 mM NAC-treated group (group E; *p<0.05). No significant differences were observed between any other two groups. **C**: The effect of NAC on the activity of Na, K-ATPase in rabbit lenses (mean±SD). Lenses exposed to hyperoxia (group B) had a significant reduction of Na, K-ATPase activity (*p<0.05). Compared with the hyperoxia-exposed group (group B), the activity of Na, K-ATPase was increased dramatically in the 20 mM and 40 mM NAC-treated groups (*p<0.05), but did not fully restore it to the normal level. No significant differences were observed between any other two groups. **D**: The effect of NAC on the activity of CAT in rabbit lenses (mean±SD). Lenses exposed to hyperoxia (group B) had a significant reduction of CAT activity (*p<0.05); Compared with the hyperoxia-exposed group (group B), the activity of CAT was increased dramatically in the 20 mM and 40 mM NAC-treated groups (*p<0.05). No significant differences were observed between any other two groups. **E**: The effect of NAC on the activity of GR in rabbit lenses (mean±SD). No significant difference was noted between groups. Group A was the control group, group B was the hyperoxia-exposed rabbit lenses group, group C-F were the hyperoxia-exposed, NAC-treated groups: 5 mM NAC (group C), 10 mM NAC (group D), 20 mM NAC (group E), and 40 mM NAC (group F).

There was a significant reduction of water-soluble protein content in the lenses of group B, compared to that in group A lenses (by 13.1%; p<0.05 Figure 4A). No statistically significant difference was found between the control group and the NAC-treated groups. The four NAC-treated groups maintained their levels of water-soluble protein. Compared with the lenses in group B, the level of water-soluble protein was significantly greater in the 20 mM NAC-treated group (by 10.6%; p<0.05) and in the 40 mM NAC-treated group (by 11.9%; p<0.05).

### Glutathione determination

Hyperoxia lowered the level of GSH significantly (for group B, compared to the control group) by 33% (p<0.05). NAC, 20 mM NAC, and 40 mM NAC, seemed to protect GSH levels by 28.2% (p<0.05) and 23.9% respectively (Figure 4B), but no significant difference was found between the 20 mM NAC- and the 40 mM NAC-treated groups.

### The activity of enzymes

The activity of Na, K-ATPase was significantly lowered (38.2%) by hyperoxia (comparing group B to group A; p<0.05). The enzymatic activity (Figure 4C) showed that hyperoxia damaged the activity of Na, K-ATPase in the lens. The reduction of Na, K-ATPase activities by hyperoxia was prevented by NAC. The Na, K-ATPase activity was protected in groups E and F (p<0.05; Figure 4C). However, there was no significant difference between groups E and F.

The results for catalase activity in the lenses after incubation are shown in Figure 4D. The activity of CAT (about 39.9% [p<0.05]) was significantly lower in hyperoxia-exposed lenses than in the control lenses. The treatment by NAC resulted in a partial protection of CAT activity, by 34.7% (p<0.05) in group E, and by 37.5% (p<0.05) in group F.

The activity of GR was decreased by 18% in the lenses of group B, and was higher in the four NAC-treated groups, compared to the untreated group, about 8.0% medially. However, there were no statistically significant differences (Figure 4E).

## Discussion

The lens is avascular, and depends on diffusion for its oxygen supply. The vitreous humor impedes the flow of oxygen from the retina, and therefore normally keeps the concentration of O_2_ at the surface of the lens low. With age, liquefaction of the human vitreous humor may damage the oxygen tension gradient in the vitreous cavity, permitting increased migration of O_2_ from the retinal circulation through the vitreous space to the posterior side of the lens. In addition, loss of protein sulfhydryl groups, oxidation of methionine residues, and damage to enzymatic antioxidant defense systems in the lens possibly accelerate the formation of nuclear cataract [34]. A study by Harocopos et al. [35] showed that the correlation between the extent of nuclear opaciﬁcation and the percent liquefaction was highly signiﬁcant, after adjusting for age. Age was also signiﬁcantly associated with nuclear opaciﬁcation. Oxidative damage to lens proteins and lipids is a hallmark of age-related cataract. Therefore, vitreous degeneration in the eye may contribute to increased oxidative stress and formation of nuclear cataract.

High oxygen tension after vitrectomy is also associated with a high risk of progressive nuclear sclerosis [3,34]. Clinical studies have reported that lens opacities progress in 41%–80% of operated eyes, after removal of idiopathic epiretinal membranes using vitrectomy [36,37]. In elderly individuals undergoing vitrectomy surgery, 60% to 98% will develop clinically significant nuclear sclerosis within two years [38]. Holekamp et al. also found that vitrectomy surgery increased intraocular oxygen tension during, and for prolonged periods after, surgery. This exposes the crystalline lens to abnormally high oxygen, and may lead to nuclear cataract formation [3]. These observations may provide an insight into the development of nuclear cataract after vitrectomy.

Additional evidence to link O_2_ with the formation of nuclear cataract comes from the therapeutic treatment of patients with hyperbaric oxygen (HBO). One study showed that of 15 patients who had clear lens nuclei before exposure to hyperbaric oxygen treatment, seven patients later showed development of nuclear cataract that decreased visual acuity; others showed increased nuclear light scattering [4]. Similar results regarding the detrimental effects of hyperbaric oxygen on the lens nucleus have also been reported in animal models. In Schocket’s [39] study, in which mice were treated with HBO, one half of the surviving animals had nuclear cataracts within eight months. Therefore, hyperoxia has been linked to the development of human nuclear cataract. As in the present study, these studies found no evidence of nuclear opacities after milder hyperoxia treatment. This may have been due to the short duration and normal oxygen pressure used for incubation. The present study only showed the lens changes induced by hyperoxia at an earlier stage.

The past clinical findings just described have led some laboratories to investigate the impact of antioxygen agents on the transparency or optical quality of the lenses of experimental animals [40]. In the present study, we investigated the preventive and ameliorating effects of NAC on lenses exposed to hyperoxia, as reﬂected by the activities of two major enzymes. One is catalase, a key enzyme that neutralizes formation of reactive oxygen species. The other is Na, K-ATPase, which is responsible for the ionic balance of the cells. In addition, the contents of GSH and the water-soluble protein were changed in this study. GSH is the most important endogenous rechargeable antioxidant. The content of water-soluble protein indicates lens damage by hyperoxia: the greater the damage to the lens, the lower the content of water-soluble protein. Based on that observation, we conclude that the observed lens opacification in the present study was the result of the high oxygen and oxidative load. The level of GSH was decreased in lenses exposed to hyperoxia, and it increased in NAC-treated groups; 20 mM NAC appeared to protect the thiols (from GSH) in four treated groups, compared to the control group. That means NAC corrected the reduction in GSH concentration.

A reduction in CAT activity in the hyperoxia-exposed group was observed. Hydrogen peroxide is one of the molecules responsible for oxidative injury to the lens. CAT is a key enzyme against relatively high concentrations of peroxide [41]. The lenses with NAC showed lower damage from catalase, which suggests that NAC results in significant protection of the activities of catalase.

ATPase activity was clearly lower in the lenses exposed to hyperoxia without NAC, when compared to the control lenses. The extracellular and intracellular cation balance is the result of permeability properties of the lens cell membranes, and of the activity of the Na, K-ATPase pump. Inhibition of Na, K-ATPase results in loss of cation balance and elevation of water content in the lens [42]. A previous study suggested that membrane permeability is increased with cataract development, and that oxidative stress can damage membrane permeability and stability [43]. Therefore, the enzyme activities decreased in the hyperoxia-exposed group. NAC has mucolytic and anticollagenolytic properties: it can keep the cell membrane and ionic pump stable [23]. The oxygen levels in this study may seem high, being four or five times the normal atmospheric level, but they are much lower than has been used in hyperbaric oxygen experiments. Furthermore, there is the usual problem: that we have to study changes within a reasonable time in the laboratory, whereas cataract in vivo takes years to form. Even after vitrectomy, it takes months.

In conclusion, oxygen plays a key role in cataract formation. It is also believed to be one of the potential causative agents for the development of nuclear cataract, following vitrectomy, and after HBO treatment [3,6,7]. The present study’s results are the first report on a possible role for NAC in the prevention of hyperoxia-induced damage to rabbit lenses. It appears that NAC attenuates oxidative lenticular damage and the protective effects of NAC against oxidative damage through its favorable effect on GSH, catalase, and Na, K-ATPase activities. The observed antioxidant protective effects of NAC could result from intra-lenticular action. Given our current state of knowledge, we are unable to specify whether this constriction has the same effects. The results of the present investigation suggest that NAC is able to signiﬁcantly retard experimental hyperoxia-induced lens damage.

## References

[1] Brian G Taylor H Cataract blindness: challenges for the 21st century. Bull World Health Organ 2001 79 249 56 11285671 PMC2566371

[2] Spector A Garner WH Hydrogen peroxide and human cataract. Exp Eye Res 1981 33 673 81 7318962 10.1016/s0014-4835(81)80107-8

[3] Simpanya MF Ansari RR Suh KI Leverenz VR Giblin FJ Aggregation of lens crystallins in an in vivo hyperbaric oxygen guinea pig model of nuclear cataract: dynamic light-scattering and HPLC analysis. Invest Ophthalmol Vis Sci 2005 46 4641 51 16303961 10.1167/iovs.05-0843 PMC1364483

[4] Palmquist BM Philipson B Barr PO Nuclear cataract and myopia during hyperbaric oxygen therapy. Br J Ophthalmol 1984 68 113 7 6691953 10.1136/bjo.68.2.113 PMC1040267

[5] Truscott RJ Age-related nuclear cataract-oxidation is the key. Exp Eye Res 2005 80 709 25 15862178 10.1016/j.exer.2004.12.007

[6] Giblin FJ Schrimscher L Chakrapani B Reddy VN Exposure of rabbit lens to hyperbaric oxygen in vitro: regional effects on GSH level. Invest Ophthalmol Vis Sci 1988 29 1312 9 3417415

[7] Barbazetto IA Liang J Chang S Zheng L Spector A Dillon JP Oxygen tension in the rabbit lens and vitreous before and after vitrectomy. Exp Eye Res 2004 78 917 24 15051473 10.1016/j.exer.2004.01.003

[8] Giblin FJ Glutathione: a vital lens antioxidant. J Ocul Pharmacol Ther 2000 16 121 35 10803423 10.1089/jop.2000.16.121

[9] Sakaue H Negi A Honda Y Comparative study of vitreous oxygen tension in human and rabbit eye. Invest Ophthalmol Vis Sci 1989 30 1933 7 2777513

[10] McNulty R Wang H Mathias RT Ortwerth BJ Truscott RJ Bassnett S Regulation of tissue oxygen levels in the mammalian lens. J Physiol 2004 559 883 98 15272034 10.1113/jphysiol.2004.068619 PMC1665185

[11] Spector A Ma W Wang RR The aqueous humor is capable of generating and degrading H2O2. Invest Ophthalmol Vis Sci 1998 39 1188 97 9620079

[12] McGahan MC Harned J Grimes AM Fleisher LN Regulation of ferritin levels in cultured lens epithelial cells. Exp Eye Res 1994 59 551 5 9492756 10.1006/exer.1994.1140

[13] Moffat BA Landman KA Truscott RJ Sweeney MH Pope JM Age-related changes in the kinetics of water transport in normal human lenses. Exp Eye Res 1999 69 663 9 10620395 10.1006/exer.1999.0747

[14] Spector A Oxidative stress-induced cataract: mechanism of action. FASEB J 1995 9 1173 82 7672510

[15] Varma SD Kumar S Richards RD Light-induced damage to ocular lens cation pump: prevention by vitamin C. Proc Natl Acad Sci USA 1979 76 3504 6 291017 10.1073/pnas.76.7.3504 PMC383855

[16] Li WC Wang GM Wang RR Spector A The redox active components H_2_O_2_ and N-acetyl-L-cysteine regulate expression of c-jun and c-fos in lens systems. Exp Eye Res 1994 59 179 90 7835407 10.1006/exer.1994.1096

[17] Li DW Spector A Hydrogen peroxide-induced expression of the proto-oncogenes, c-jun, c-fos and c-myc in rabbit lens epithelial cells. Mol Cell Biochem 1997 173 59 69 9278255 10.1023/a:1006828402225

[18] Lou MF Redox regulation in the lens. Prog Retin Eye Res 2003 22 657 82 12892645 10.1016/s1350-9462(03)00050-8

[19] Ganea E Harding JJ Glutathione-related enzymes and the eye. Curr Eye Res 2006 31 1 11 16421014 10.1080/02713680500477347

[20] Bhuyan KC Bhuyan DK Regulation of hydrogen peroxide in eye humors. Effect of 3-amino-1H–1,2,4-triazole on catalase and glutathione peroxidase of rabbit eye. Biochim Biophys Acta 1977 497 641 51 889879 10.1016/0304-4165(77)90284-7

[21] Delamere NA Tamiya S Expression, regulation and function of Na, K-ATPase in the lens. Prog Retin Eye Res 2004 23 593 615 15388076 10.1016/j.preteyeres.2004.06.003

[22] Paterson CA Delamere NA ATPases and lens ion balance. Exp Eye Res 2004 78 699 703 15106949 10.1016/j.exer.2003.09.018

[23] Kelly GS Clinical applications of N-acetylcysteine. Altern Med Rev 1998 3 114 27 9577247

[24] Jain AK Lim G Langford M Jain SK Effect of high-glucose levels on protein oxidation in cultured lens cells, and in crystallin and albumin solution and its inhibition by vitamin B6 and N-acetylcysteine: its possible relevance to cataract.formation in diabetes. Free Radic Biol Med 2002 33 1615 21 12488130 10.1016/s0891-5849(02)01109-7

[25] Shattuck KE Rassin DK Grinnell CD N-acetylcysteine protects from glutathione depletion in rats exposed to hyperoxia. JPEN J Parenter Enteral Nutr 1998 22 228 33 9661124 10.1177/0148607198022004228

[26] Zhang S Chai FY Yan H Guo Y Harding JJ Effects of N-acetylcysteine and glutathione ethylester drops on streptozotocin-induced diabetic cataract in rats. Mol Vis 2008 14 862 70 18490958 PMC2386505

[27] Liu XC Wang P Yan H A rabbit model to study biochemical damage to the lens after virectomy: effects of N-acetylcysteine. Exp Eye Res 2009 88 1165 70 19450459 10.1016/j.exer.2009.01.001

[28] Rathbun WB Nagasawa HT Killen CE Prevention of naphthalene-induced cataract and hepatic glutathione loss by the L-cysteine prodrugs, MTCA and PTCA. Exp Eye Res 1996 62 433 41 8795461 10.1006/exer.1996.0048

[29] Geraldine P Sneha BB Elanchezhian R Ramesh E Kalavathy CM Kaliamurthy J Thomas PA Prevention of selenite-induced cataractogenesis by acetyl-L-carnitine: an experimental study. Exp Eye Res 2006 83 1340 9 16962580 10.1016/j.exer.2006.07.009

[30] Reddy VN Varma SD Chakrapani B Transport and metabolism of glutathione in the lens. Exp Eye Res 1973 16 105 14 4720448 10.1016/0014-4835(73)90305-9

[31] Secchi GC Ambrosi L Rezzonico A Studies on Na, K-ATPase and the acetylcholinesterase activity in erythrocyte membranes in saturnine anemia. Med Lav 1968 59 593 8 4237685

[32] Beers RF Jr Sizer IW A spectrophotometric method for measuring the breakdown of hydrogen peroxide by catalase. J Biol Chem 1952 195 133 40 14938361

[33] Bergmeyer HU. Methods of Enzymatic Analysis. New York: Academic Press 1963; 1064.

[34] Holekamp NM Shui YB Beebe DC Vitrectomy surgery increases oxygen exposure to the lens: a possible mechanism for nuclear cataract formation. Am J Ophthalmol 2005 139 302 10 15733992 10.1016/j.ajo.2004.09.046

[35] Harocopos GJ Shui YB McKinnon M Holekamp NM Gordon MO Beebe DC Importance of vitreous liquefaction in age-related cataract. Invest Ophthalmol Vis Sci 2004 45 77 85 14691157 10.1167/iovs.03-0820

[36] Cherfan GM Michels RG de Bustros S Enger C Glaser BM Nuclear sclerotic cataract after vitrectomy for idiopathic epiretinal membranes causing macular pucker. Am J Ophthalmol 1991 111 434 8 2012145 10.1016/s0002-9394(14)72377-3

[37] Smiddy WE Michels RG Glaser BM deBustros S Vitrectomy for macular traction caused by incomplete vitreous separation. Arch Ophthalmol 1988 106 624 8 3358728 10.1001/archopht.1988.01060130678025

[38] Cheng L Azen SP El-Bradey MH Scholz BM Chaidhawangul S Toyoguchi M Freeman WR Duration of vitrectomy and postoperative cataract in the vitrectomy for macular hole study. Am J Ophthalmol 2001 132 881 7 11730653 10.1016/s0002-9394(01)01263-6

[39] Schocket SS Esterson J Bradford B Michaelis M Richards RD Induction of cataracts in mice by exposure to oxygen. Isr J Med Sci 1972 8 1596 601 4647826

[40] Schaal S Beiran I Bormusov E Chevion M Dovrat A Zinc-desferrioxamine reduces damage to lenses exposed to hyperbaric oxygen and has an ameliorative effect on catalase and Na, K-ATPase activities. Exp Eye Res 2007 84 455 63 17174302 10.1016/j.exer.2006.10.019

[41] Ma W Li D Sun F Kleiman NJ Spector A The effect of stress withdrawal on gene expression and certain biochemical and cell biological properties of peroxide-conditioned cell lines. FASEB J 2004 18 480 8 15003993 10.1096/fj.03-0732com

[42] Huang WH Wang Y Askari A Na, K-ATPase: inactivation and degradation induced by oxygen radicals. Int J Biochem 1992 24 621 6 1325381 10.1016/0020-711x(92)90337-z

[43] Yan H Harding JJ Xing K Lou MF Revival of glutathione reductase in human cataractous and clear lens extracts by thioredoxin and thioredoxin ductase, in conjunction with α-crystallin or thioltransferase. Curr Eye Res 2007 32 455 63 17514531 10.1080/02713680701257837

